# ISDD: A computational model of particle sedimentation, diffusion and target cell dosimetry for *in vitro *toxicity studies

**DOI:** 10.1186/1743-8977-7-36

**Published:** 2010-11-30

**Authors:** Paul M Hinderliter, Kevin R Minard, Galya Orr, William B Chrisler, Brian D Thrall, Joel G Pounds, Justin G Teeguarden

**Affiliations:** 1 Biological Sciences Division, Pacific Northwest National Laboratory, Richland WA, 99352. USA; 2 Environmental and Molecular Sciences Laboratory, Pacific Northwest National Laboratory, Richland, WA, 99352. USA; 3 B-FAST, Battelle Center for Fundamental and Applied Systems Toxicology, USA

## Abstract

**Background:**

The difficulty of directly measuring cellular dose is a significant obstacle to application of target tissue dosimetry for nanoparticle and microparticle toxicity assessment, particularly for *in vitro *systems. As a consequence, the target tissue paradigm for dosimetry and hazard assessment of nanoparticles has largely been ignored in favor of using metrics of exposure (e.g. μg particle/mL culture medium, particle surface area/mL, particle number/mL). We have developed a computational model of solution particokinetics (sedimentation, diffusion) and dosimetry for non-interacting spherical particles and their agglomerates in monolayer cell culture systems. Particle transport to cells is calculated by simultaneous solution of Stokes Law (sedimentation) and the Stokes-Einstein equation (diffusion).

**Results:**

The *In vitro *Sedimentation, Diffusion and Dosimetry model (ISDD) was tested against measured transport rates or cellular doses for multiple sizes of polystyrene spheres (20-1100 nm), 35 nm amorphous silica, and large agglomerates of 30 nm iron oxide particles. Overall, without adjusting any parameters, model predicted cellular doses were in close agreement with the experimental data, differing from as little as 5% to as much as three-fold, but in most cases approximately two-fold, within the limits of the accuracy of the measurement systems. Applying the model, we generalize the effects of particle size, particle density, agglomeration state and agglomerate characteristics on target cell dosimetry *in vitro*.

**Conclusions:**

Our results confirm our hypothesis that for liquid-based *in vitro *systems, the dose-rates and target cell doses for all particles are not equal; they can vary significantly, in direct contrast to the assumption of dose-equivalency implicit in the use of mass-based media concentrations as metrics of exposure for dose-response assessment. The difference between equivalent nominal media concentration exposures on a μg/mL basis and target cell doses on a particle surface area or number basis can be as high as three to six orders of magnitude. As a consequence, *in vitro *hazard assessments utilizing mass-based exposure metrics have inherently high errors where particle number or surface areas target cells doses are believed to drive response. The gold standard for particle dosimetry for *in vitro *nanotoxicology studies should be direct experimental measurement of the cellular content of the studied particle. However, where such measurements are impractical, unfeasible, and before such measurements become common, particle dosimetry models such as ISDD provide a valuable, immediately useful alternative, and eventually, an adjunct to such measurements.

## Background

The rapid pace of introduction of new nanomaterials into commerce, rising human exposure through consumer products, and the absence of reliable safety data or exposure regulations [1] have raised risk assessment to a top priority for stakeholders in the allied nanomaterial fields [2,3]. Complete animal-based safety testing of the virtually limitless number of potential engineered nanoparticles (ENPs) and derivative microscale particles is widely recognized as fiscally and temporally impossible. There are however, promising data suggesting that the biological response to ENPs can, for specific classes of materials, be related to their structural and physicochemical properties[4-6], as has been the case for classes of chemicals and pharmaceuticals such as PCBs, Dioxins, and the statin drugs [7-9]. This work indicates there is an opportunity for a risk assessment paradigm for ENPs that parallels the National Research Council's (NRC) vision for toxicology in the 21^st ^century: the use of selected *in vitro *assays in cell culture systems for hazard screening and development of quantitative structure activity models, limited animal studies for understanding kinetics, and the use of pharmacokinetic models for extrapolation of results from *in vitro *to *in vivo*, between species and across sensitive populations[10]. ENPs pose unique challenges to the NRC paradigm[11], particularly in the area of dosimetry for *in vitro *systems[12], where the science lags, and for extrapolation to and between rodent test species and humans. Thus, there is a critical need to develop a dosimetry-enabled framework for ENP risk and hazard assessment.

The dose-response paradigm for the fields of pharmacology and toxicology are predicated on the principle that response is proportional to the concentration of the effector molecule at the site of action [13]. The use of target tissue dose, rather than less specific measures of "dose" such as exposure or administered dose, improves correlations between dose and response for drugs, chemicals, and inhaled gases and particles[14-17]. Target tissue dose has become the gold standard for dose-response assessment in pharmaceutical safety assessment and chemical risk assessment[18], and was most recently used by National Institute for Occupational Safety and Health (NIOSH) to conduct a risk assessment for nanoscale TiO_2_[19].

Surprisingly, despite wide use of *in vitro *systems for toxicity assessment of ENPs, the target tissue paradigm for dosimetry has largely been ignored in favor of using metrics of exposure, principally ENP concentration on a mass, number, or surface area basis. Use of exposure metrics, which are not reliable measures or proxies of target cell dose across particle size or systems, may be one root cause of the failure of *in vitro *systems to predict *in vivo *response, as reported by Sayes et al. [20], and Warheit et al. [21]. Other factors, for example pharmacodynamic differences or limitations of using a single cell system to represent the integrated function of a tissue, are also plausible confounders for *in vitro*-*in vivo *predictions. Nonetheless, without a consistent, biologically relevant measure of dose to compare responses across systems, *in vitro *systems cannot be expected to represent dose-responses *in vivo*.

**Figure 1:**
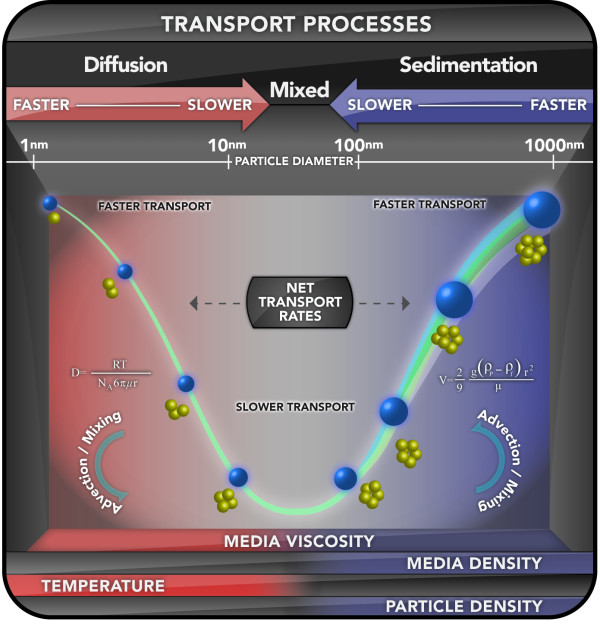
**Important Particle Transport Processes for In Vitro Systems**. Depiction of the important processes and system characteristics affecting particle transport rates in liquid containing *in vitro *systems. Transport of particles ≤ ~10 nm is controlled principally by diffusion, and can become relatively fast at particle sizes less than 10 nm. Transport of particles greater than ~200 nm can also be relatively fast, particularly for dense particles like the metals, and is controlled by sedimentation. Slower transport is expected to occur between 10 and 100 nm, where both diffusion and sedimentation together control transport, but neither process is particularly effective.

The definition of dose for nanoparticles in an *in vitro *system is more dynamic, more complicated, and less comparable across particle types than it is for soluble chemicals. Particles settle, diffuse and agglomerate at rates that differ in relation to their size, density, and surface physicochemistry (reviewed in [12])(Figure 1). These particle properties are expected to significantly affect cellular dose, but are largely ignored in the conduct of *in vitro *nanomaterial toxicity studies. Limbach raised this issue [4], presenting experimental evidence that transport to cells of 25-50 nm and 250-500 nm cerium oxide particles are different, depending in the former case on diffusion and the latter case on gravitational settling. This differential transport was shown to affect cellular uptake rates [4] and perhaps toxicity [12,22]. Extending their analysis to include differences in transport rates revealed that indeed particle dependent differences in transport to cells from settling and diffusion could significantly affect relative toxicity [12].

Most *in vitro *studies with nanomaterials would benefit from direct measures of cellular dose. However, experimental measurement of cellular dose *in vitro *(and *in vivo*) is often difficult or costly, and as such, is a considerable limitation of *in vitro *studies. Thus, measures of target cell dose will often not be available to risk assessors who must interpret published studies that report particle characteristics and biological effects, but not measures of cellular dose.

The dynamics of particles in liquids are well studied and mathematical approaches for describing both diffusion and gravitational settling have been developed [23]. These approaches have yet to be formed into an approach for describing the particokinetics--the combined influence of diffusion and gravitational settling on particle transport to cells *in vitro*. We have developed a computational model of particokinetics (sedimentation, diffusion) and dosimetry for non-interacting spherical particles and their agglomerates in a common cell culture system. ISDD is an *in vitro *counterpart to the Multipath Particle Deposition Model (MPPD) for inhaled particles[24]. The model is developed from first principles--Stokes sedimentation and Stokes-Einstein diffusion--and verified against experimentally measured rates of nano- and micro-particle transport across several particles sizes, and densities as well as agglomerates.

ISDD provides the first computational *in vitro *target cell dosimetry platform to improve the accuracy and predictive power of *in vitro *systems for assessing ENP hazard, and significantly improve the ability of researchers to design *in vitro *studies based on doses that directly relate to probable human exposures based on target tissue cell/dose comparisons. The model overcomes the current absence of information on the comparative rates and extent (e.g. dose) of nano- and micro-scale particle transport to cells in culture.

## Materials and methods

### Model Development and Evaluation

#### Model Overview

In standard liquid-based cell culture systems, the amount of particles associated with cells at any time is a function of the rate of delivery of particles to the cells, how strongly particles adhere to the cell surface, and the rates of cellular uptake and loss by degradation or exocytosis.

ISDD applies well established, long-used principles of diffusional and gravitational transport of particles in viscous media to calculate the movement of particles from the media to the bottom of a vessel where cells reside. The net rate of transport downward toward the bottom of the vessel is calculated within a single partial differential equation, which is solved numerically to calculate the fraction of material transported from media to the bottom of the vessel. Simulations are conducted using commonly available inputs for monodisperse particles: temperature, media density and viscosity, media height, hydrodynamic particle size in the test media, and particle density. Simulations of agglomerates also require two additional parameters describing how the primary particles are packed to form the agglomerate. The model produces a time-course of particle surface area, number and mass transported to the bottom of the vessel, referred to as the delivered dose, which can be compared to measured values in a cell free environment. The delivered dose can also be compared to measured amounts associated with cells (in or adhered to), which is an appropriate, but possibly less certain comparison because the roles of cellular uptake, adherence, and loss of adhered material during washing are not accounted for explicitly in the current formulation of ISDD. ISDD focuses on particle transport because this process can be rate limiting, is very valuable for the experimentalist to understand, can be simulated with a relatively small set of easy to access parameters, and is independent of cell type and other experimental conditions that affect cellular uptake. Moreover, at this time, it is experimentally difficult to separate particle uptake (particles in a cell) from cell associated particles (on a cell or in a cell). If necessary, modifications to the boundary conditions or assumptions regarding fractional uptake can be used to account for cellular uptake. Thus, ISDD calculates the delivered dose, which is equivalent to particles associated with the cell (on a cell or in a cell), the only commonly available experimental measure of target cell dose.

#### Derivation and Description of ISDD

The general dynamics of particles in viscous media are well studied [25], and the mathematical basis for describing particle transport in liquids has been available for more than 100 years [26]. Three primary processes transport particles in static (non-flowing) uniform solutions comprising the majority of liquid-containing cell culture systems: diffusion, sedimentation, and advection (transfer by motion of the fluid) [27]. In the formulation of ISDD, we made the reasonable assumption that advective forces in cell culture medium held at constant temperature, without disturbance, are minimal and do not significantly affect particle transport. More practically, diffusion and sedimentation rates can be calculated from commonly measured particle, culture medium, and experimental design characteristics while the tools for experimentally measuring advective forces on particles are not expected to be widely available in biology labs. Particles, primary or agglomerates, are assumed to be independent and non-interacting (e.g. agglomerates do not form during the simulation). The sides of the cell culture dish are not considered because at common media heights, the surface area of the sides is very small relative to the bottom. Large errors in model predictions would be evidence that one or more of these simplifying assumptions may be wrong.

Stokes' law defines the gravitationally driven sedimentation rate (V, m/s) of particles in solution from the viscosity (μ, Pa·s) of the media, the relative densities (kg/m^3^) of the particle (ρ_p_) and fluid (ρ_f_), the diameter of the particle (d, m) and the acceleration due to gravity (g, m/s^2^):

(1) $$\text{V}=\frac{\text{g}\left({\text{ρ}}_{\text{p}}-{\text{ρ}}_{\text{f}}\right)\;\;{\text{d}}^{\text{2}}}{\text{18}\mu }$$

The Stokes-Einstein equation describes the relationship between particle diameter and the rate of diffusion (D, m^2^/s) as a function of viscosity and temperature (T, °K):

(2) $$\text{D}=\frac{\text{RT}}{{\text{3N}}_{\text{A}}\pi \mu \;\text{d}}$$

where R is the gas constant (L·kPa/K/mol) and N_A _is Avogadro's number.

The movement of particles through a fluid can also give rise to fluid motion and turbulence. Generally, fluid flow can be described by the Reynolds number, the dimensionless ratio of inertial to viscous forces. Provided that the Reynolds number is below one, equations 1 and 2 define the only necessary terms in the convection-diffusion equation for laminar flow [28]. Reynolds numbers for spheres less than 100 µm are less than one [29]. Reynolds numbers were more than an order of magnitude less than one for all particles, including agglomerates, considered in this manuscript.

Mason and Weaver derived a mathematical solution to the laminar convection-diffusion equation, a parabolic partial differential equation (PDE) [23]:

(3) $$\frac{\partial \text{n}}{\partial \text{t}}=\text{D}\frac{\partial^{\text{2}}\text{n}}{\partial {\text{x}}^{\text{2}}}-\text{V}\frac{\partial \text{n}}{\partial \text{x}}$$

where t and x are the time (s) and distance (m) dimensions, D and V are the previously defined diffusivity and sedimentation velocity (equations 1 and 2), and n is the particle concentration. The particle concentration may be defined in any convenient units such as particle number/mL or grams/mL. The first term on the right hand side of equation 3 describes the particle motion by diffusion; the second describes the sedimentation.

For initial conditions we assume a uniform particle distribution at initiation of the experiment (equation 4a). The boundary conditions are, a) no particle flux across the top of the media (equation 4b), and b) zero concentration at the bottom (equation 4c), equivalent to a condition where particles reaching the bottom and adhering to cells no longer affect particle flux or concentration calculations (L = total media height):

(4) $$\begin{array}{l} \text{a:}\;{\text{n}}_{\text{0}}=\text{constant for}\;\text{all}\;\text{x,}\;\text{t}=\text{0}\\ \text{b:}\;\text{D}\frac{\partial \text{n}}{\partial \text{x}}=\text{Vn at}\;\text{x}=\text{L}\;\text{(top)}\\ \text{c:}\;\text{n}=\text{0 at}\;\text{x}=\text{0}\;\text{(bottom)} \end{array}$$

These boundary conditions can be modified for consistency with experimentally measured boundary conditions (e.g. non-uniform distribution of particles at the start of an experiment, or particles that do not adhere to cells at the bottom). Thus, the *in vitro *dosimetry model is a partial differential equation for dynamically simulating the transport of micro and nanoparticles in suspension in the vertical dimension (parallel to gravity).

To enable an efficient and stable computational solution, equation 3 was transformed into a dimensionless form using the following dimensionless variables:

(5) $$\bar{\text{x}}=\frac{\text{x}}{\text{L}}\text{,}\;\bar{\text{ t }}=\frac{\text{tV}}{\text{L}}\text{,}\;\text{α}=\frac{\text{D}}{\text{VL}}\text{,}\;\bar{\text{ n }}=\frac{\text{n}}{{\text{n}}_{\text{0}}}$$

which yields the transformed PDE and boundary conditions used in the model code (a-c):

(6) $$\begin{array}{l} \frac{\partial \bar{\text{n}}}{\partial \bar{\text{t}}}=\alpha \frac{\partial^{\text{2}}\bar{\text{n}}}{\partial {\bar{\text{x}}}^{\text{2}}}-\frac{\partial \bar{\text{n}}}{\partial \bar{\text{x}}}\\ \text{a:}\;\bar{\text{n}}=\text{1 for}\;\text{all }\;\bar{\text{x}},\bar{\text{t}}=\text{0}\\ \text{b:}\;\alpha \frac{\partial \bar{\text{n}}}{\partial \bar{\text{x}}}=\bar{\text{n}}\text{ at }\;\bar{\text{x}}=1\;\text{(top)}\\ \text{c:}\;\bar{\text{n}}=\text{0 at }\;\bar{\text{x}}=\text{0}\;\text{(bottom)} \end{array}$$

The solution to equation 6 provides the means to directly calculate the net movement of particles of different size and density in liquid media, i.e. cell culture medium, to cells at the bottom of in an *in vitro *test system. ISDD outputs the fraction, total number, surface area and or mass of particles reaching cells at a given time, which can be directly compared to measured values in a cell free system (reaching the bottom of a dish) or measured values of cell associated material (adhered to or within cells). Along with input functions for parameter values, equation 6 constitutes the model for monomers. ISDD was developed in Matlab^® ^(MathWorks, Inc.), and is solved numerically using the PDE solver in Matlab^®^. The model is available from the authors upon request.

Most nanoparticles exist in some degree of agglomeration in cell culture medium [30]. Agglomeration affects particle shape, density and size, with corresponding effects on both diffusion and sedimentation [12,30]. Because agglomerates are not necessarily composed of efficiently packed particles, agglomerates are modeled as having a fractal structure according to Sterling et al [31]. The interparticle pore space in fractal agglomerates comes from two sources: packing effects and the fractal nature of the aggregate [31]. Both account for the entrapment of media between particles in the agglomerate (i.e. porosity) and the resulting reduction in density. Packing effects are determined by the shapes of the monomers and how they are packed into the agglomerate. The fractal nature is determined by the flocculation processes causing formation of the agglomerate [31]. The fractal nature of the agglomerate, represented by the fractal dimension (1< DF <3), is generally more important in determining density and porosity than the packing factor (0< PF <1)[31]. Sterling used this fractal description to effectively model the flocculation and sedimentation of clay and colloidal silica agglomerates.

A fractal dimension of 3 reflects a perfect sphere with little or no fractal structure and a porosity of zero (no entrapped liquid). Similarly, a PF of 1 reflects the absence of pore space in the agglomerate. In the absence of an experimentally measured PF, a value of 0.637 for randomly packed spherical monomers reported by Sterling was used [31]. The number of single particles per agglomerate (Np), agglomerate porosity (ε_agg, _unitless) and agglomerate density (ρ_agg_, kg/m^3^) are calculated from the experimentally measured value of the agglomerate diameter in media (d_agg_), and the primary particle size and density, as described by Sterling: [31]:

(7) a: dagg=d (NpPF)1DFb: εagg=1−(daggd)DF−3c: ρagg=(1−εagg) ρp+εaggρf

The agglomerate sedimentation velocity can then be related to the difference in density between the agglomerate and the media, as described by Sterling's equation 15[31]:

(8) $${\text{V}}_{\text{agg}}=\frac{\text{g}\left(\rho_{\text{agg}}-\rho_{\text{f}}\right)\;\;}{\text{18}\;\mu }{\text{d}}^{\text{3}-\text{DF}}{\text{d}}_{\text{agg}}^{\text{DF}-1}$$

This formulation of the sedimentation velocity equation reflects the assumption that liquid is entrapped in the agglomerate pore space and that media does not flow through the particle as it settles. The agglomerate sedimentation velocity can be substituted into the convection diffusion equation [31] (Equation 6) and solved as previously described (also using d_agg _to calculate diffusivity in Equation 2). This form of ISDD represents the agglomerate simulation code. Like the monomer code, it comprises a single PDE with supporting input functions. Thus, ISDD accommodates simulating transport of particles and agglomerates of a single size or as size class distribution, as is typically reported by dynamic light scattering (DLS) measurement.

### Media Density and Viscosity

Viscosity measurements were performed using a Cannon-Fenske opaque (reverse-flow) viscometer (Cannon Instruments, State College, PA). Samples of Gibco DMEM + GlutaMax™ (DMEM+G) media containing between 0-10% percent fetal bovine serum were placed in the viscometer and allowed to come to room temperature for approximately 10 minutes. The kinematic viscosity was calculated by multiplying the efflux time in seconds by the viscometer constant. Samples were analyzed in quadruplicate. Dynamic viscosity (used in the model) was calculated by dividing kinematic viscosity (measured) by the media density (1.0 g/mL).

The density of DMEM+G media containing between 0-10% percent serum was determined by dividing the weight of 100 mL (volumetric flask) of media by its volume. Samples were analyzed in quadruplicate.

### Particles

ISDD was verified against experimentally measured particle transport data for three different particles (polystyrene, iron oxide, silica) from three independent studies utilizing particles of different density, size and agglomeration state. Each study used a different method for quantifying particle transport (see Kinetic Data). This approach limits the potential for method-dependent bias. Superparamagnetic iron oxide nanoparticles with a manufacturer reported diameter of 30 nm (20 nm core by transmission electron microscopy (TEM)), with ~10 nm polymer coating) were obtained from Ocean Nanotechnologies (Springdale, AS). Particle size was verified by Dynamic Light Scattering (DLS) in MilliQ water and RPMI media. DLS sizing was conducted using a custom built high-sensitivity DLS instrument, enabling size-class determination at low particle concentrations (10 μg/mL) similar to those used in *in vitro *experiments. The instrument is a modification of the instrument developed by our team [32], and its accuracy was verified against polystyrene beads (Polysciences Inc, Warrington PA, Cat# 16905). The original instrument was enhanced by introducing optic fibers and avalanche photodiodes to improve the collecting efficiency.

Carboxylated fluorescent polystyrene spheres with manufacturer reported diameters of 24, 100, 210, 500 and 1100 nm in diameter were obtained from Invitrogen/Molecular Probes. Particles were virtually monodisperse in our experimental system (fluorescence microscopy, see Particokinetic Data) and could be described according to their reported primary particle size.

The amorphous silica nanoparticles used by Lison et al. [33] to generate the cellular dose data simulated here had a reported particle size of 29.3 nm (TEM) and a hydrodynamic diameter of 34.8 nm in the study media, DMEM.

### Particokinetic Data

#### Polystyrene Transport Rate Measurements

The time course of fluorescent polystyrene particles reaching the bottom of 35 mm cell culture dishes (no cells) was measured using time-lapse fluorescence microscopy (Axiovert, Zeiss) at TIRF configuration, with 100× objective lens and 2× relay lens, leading to 200× total magnification. Argon laser (Innova, Coherent) and a nitrogen cooled CCD camera (Roper Scientific) were used to excite the particles at 488 nm and acquire the emission at 510-530 nm, respectively. Culture dish bottoms were coated with Poly-L-lysine to generate a positively charged layer to which the negatively charged carboxylic acid surface modified polystyrene particles would adhere to. For each particles size, 3 mL of temperature equilibrated media containing 3.7 × 10^8 ^particles/mL was added to a dish and after a small delay (~30 seconds) the culture dish was placed in the temperature controlled (37°C) microscopy chamber and sequential images were collected every 0.5 seconds for 500 seconds. The total number of particles transported in the visual field of the microscope, 3,717 × 10^-5 ^cm^2^, was manually counted. The simulated number of particles was obtained by multiplying the calculated (ISDD) fraction of particles settled by the total number of particles in the volume of media above the counting surface (Concentration (3.7 × 10^8 ^particles/mL) × media height (0.31 cm) × deposition surface area (3,717 × 10^-5 ^cm^2^)): 4.26 × 10^3 ^particles

#### Iron Oxide Agglomerate Transport Rate Measurements

The rate of transport of large super-paramagnetic iron-oxide particles to cultured RAW 264.7 macrophages was measured under routine cell culture conditions (60 mm plates, 3 mL serum free media, 37°C, subconfluent). RAW 264.7 cells were seeded on 60 mm culture plates and incubated overnight at 37°C in RPMI media supplemented with penicillin/streptomycin, 10% fetal bovine serum, and L-Glutamine to reach estimated 80% confluency. Media was then aspirated and cells were exposed to 3 mL of serum-free media containing 2 μg/mL of iron oxide nanoparticles. To ensure uniform mixing and limit thermal convection, all exposure media was first sonicated at 37°C for 10 minutes. Immediately after the addition of dosing media, all cell cultures were gently returned to the incubator. After 2, 4, and 8 hrs the media was removed, cells were washed three times, and harvested. For each exposure time, harvested cells from three plates provided triplicate samples for dosimetry analysis, and one plate was utilized for counting. Cellular iron oxide content was measured using a custom-built magnetic particle detector. The detector itself is analogous to previous designs, and generally exploits the nonlinearity of nanoparticle magnetization [34]. Because of its high sensitivity and ease of use, this type of detector is of increased interest for different bioassays that utilize magnetic labeling strategies [35-37]. The same basic detection technology also serves as the basis for a new imaging approach for measuring the amounts of superparamagnetic label at each location within living tissue [38,39]. Key to the current application is the detection method's high linearity (between measured signal and nanoparticle amounts), and the fact that detection results are independent of either tissue-type or suspension media [40]. To calibrate our instrument a standard curve was generated using the serial dilution of stock nanoparticle suspensions and dosimetry data was normalized to measured cell numbers to give the mass of delivered nanomaterial per cell.

#### Published Silica Transport Data

Lison et al. [33] applied varying amounts (experiment A, Figure Five A of their paper) or varying concentrations (experiment B, Figure Five B) of 35 nm (DLS) spherical amorphous silica to J774 cells cultured in 0.81 cm^2 ^wells (48 well plate). Both experiments exposed cells to 90, 180, 270, 360 or 450 μL of media containing a constant particle concentration, 37 μg/mL (experiment A) or a constant particle mass, 16.7 μg (experiment B). The calculated corresponding media heights were 1.1, 2.2, 3.3, 4.4 and 4.5 mm. The media contained 10% fetal calf serum.

### ISDD Simulations of Particle Transport

ISDD was exercised in accordance with the physical characteristics of the experimental system and the particles. Measured particle hydrodynamic diameters, media heights, temperature (37°C), media density (1.0 g/cm^3^), viscosity and particle concentrations were used. No model parameters were varied to fit experimental data on monodisperse particles (silica and polystyrene). The fractal dimension (DF, Equation 7), the parameter describing how efficiently the primary particles fill the volume occupied by the agglomerate, was the only unknown parameter for the simulation of iron oxide agglomerates. Improbable values of DF, 1 (representing a rod) and 3 (representing a perfectly filled sphere) were not considered. Values near those reported for cerium oxide and fumed silicon dioxide particles of "around 2" [4,41] were varied to evaluate model behavior against the experimental data.

The following parameters were the same for all simulations: Temperature, 310°K; media density, 1.0 g/cm^3^; the media viscosity was 0.00069 Pa·s for all simulations accept the simulation of the Lison et al. silica data [32], which used a value of 0.00074 Pa·s to reflect the presence of serum proteins. Particle densities: polystyrene, 1.05 g/cm^3^; amorphous silica, 2.2 g/cm^3^); titanium dioxide, 4.23 g/cm^3^; iron oxide, 5.2 g/cm^3^; gold, 19.32 g/cm^3^. For agglomerates only: fractal dimension, 2.0-2.4, packing factor 0.637. Particle sizes were those reported in the material and methods section or in the results section. Parameter values varied in other simulations are provided in the appropriate figure legends.

## Results

### Media Characteristics

Media density was measured at different percentages of serum so most standard cell culture systems could be modeled in accordance with actual rather than assumed media density. The density of media containing 0, 1, 2, 3, 4, 5, 6, 7, 8, 9, and 10% serum was measured. There was no measureable increase in density with increasing percent serum. The average media density across all serum concentrations was 1.007 g/mL with a standard error of 0.0001 g/mL and a range of 1.006-1.009 g/mL.

Media dynamic viscosity did change, however slightly, as a function of serum concentration. DMEM+G has a viscosity of 0.9598 Pa·s. DMEM+G with 10% serum has a density of 1.011 Pa·s, approximating the viscosity of water at room temperature (~ 1.0 Pa·s at 20°C). The difference between the viscosity of water and media containing serum proteins was minimal (5%), indicating that the dynamic viscosity of water, adjusted for temperature (37°C for cell culture systems), can be used for purposes of modeling particle transport in non-serum containing media, with a modest increase of 5% for media containing 10% serum.

### Particle Characteristics

**Figure 2:**
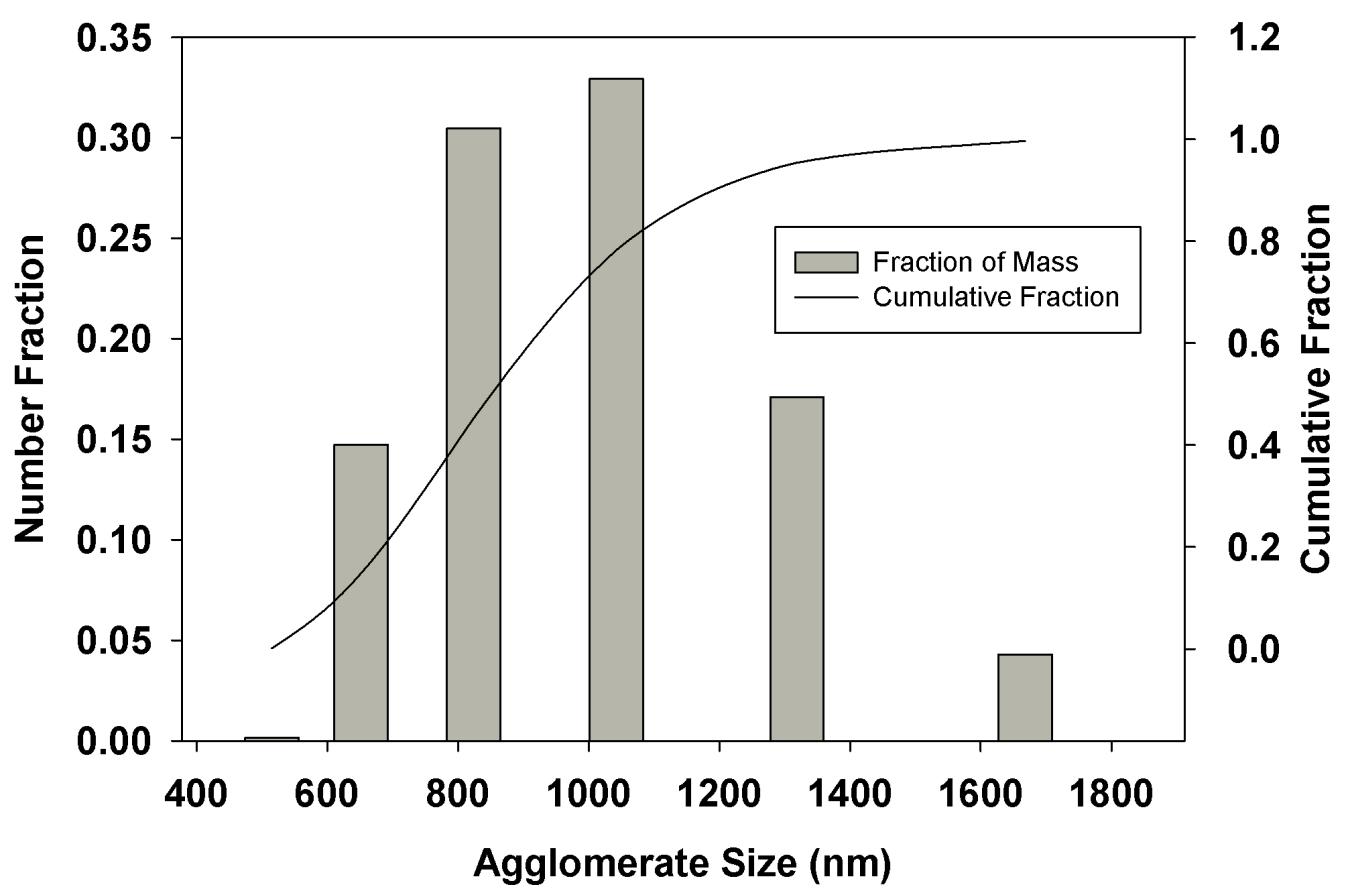
**Size Class Distribution Data for Iron Oxide Particles**. Size class distribution of iron oxide particles measured by high sensitivity DLS. Columns represent number fractions. The line represents the cumulative number fraction.

The iron oxide particles had a hydrodynamic diameter of 34.8 nm in MilliQ water, consistent with the manufacturers reported nominal diameter of ~30 nm. In RPMI media, the particles (10 μg/mL) were agglomerated and polydisperse, with average agglomerate size of 993.7 (Std = 272.1). The size class distribution is presented in Figure 2. The amorphous silica nanoparticles used by Lison et al. [33] to generate the cellular dose data simulated here had a reported particle size of 29.3 nm (TEM) and a hydrodynamic diameter of 34.8 nm in the study media, DMEM.

### Model Evaluation

Parameter values used for ISDD simulations are provided in the methods section.

#### Diffusive transport of amorphous silica

**Figure 3:**
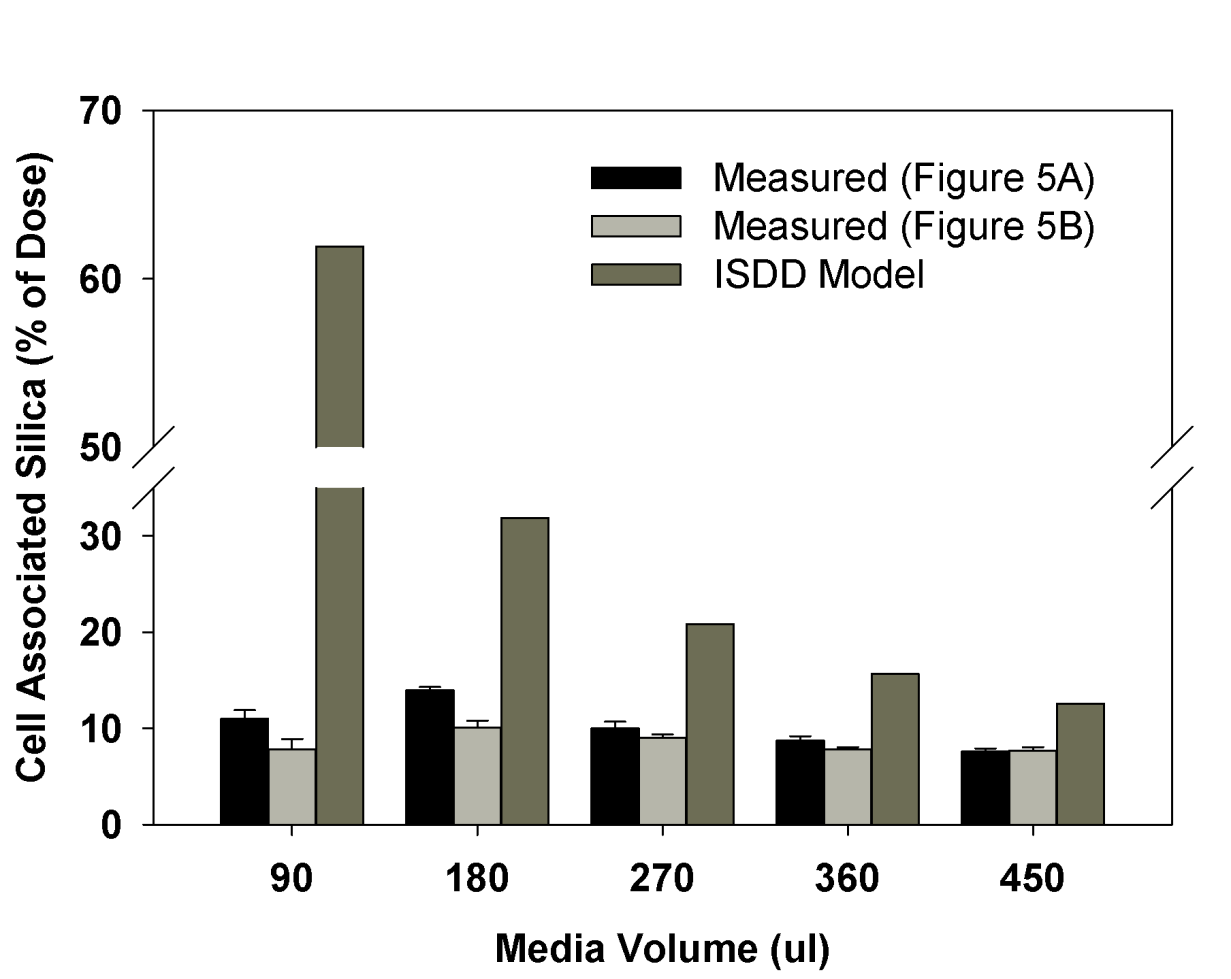
**Simulated Silica Dose**. Comparison of observed and ISDD simulated fraction of administered dose associated with cells in culture exposed to varying amounts and concentrations of 35 nm amorphous silica nanoparticles in media with heights varying from 1.1-4.5 mm. Gray bars represent measured values reported in two experiments reported by Lison et al. (2008)[32] in their Figure 5A and 5B.

To test ISDD for the diffusion driven region of the size vs. transport curve, we compared ISDD calculated percent of the administered dose delivered to cells in culture to the measured percent of administered dose associated with cells after six hours exposure followed by washing of cells [33] (Figure 3). In all cases ISDD predicted a larger fraction of the administered dose associated with the cells than was observed. Nonetheless, for most experimental conditions, there was good agreement between modeled and observed cell associated silica: the difference between modeled and observed silica was approximately two-fold, which may reflect the difference between delivered silica and silica remaining post washing, incomplete confluency of the cell monolayer, or low media volume effects on cellular function, particularly uptake of particles. The greatest difference between measured and calculated cell associated silica was, approximately six-fold, was observed for the low volume experiments where the media height was small (1.1 mm). Given the expectation that the transport processes are the same in all the experiments, it is not immediately clear why there is poorer agreement between measured and calculated silica doses for the 90 μL volume experiments. It is plausible that the more limited buffering capacity of the lower volume affected cellular function, particularly the ability to phagocytose particles. Alternatively, the more rapid build up of secreted factors (e.g. cytokines) in the smaller media volume could also impact cellular uptake of particles in a non-intuitive fashion through feedback loops. It is noteworthy that ISDD predicts a steadily increasing amount of particles as media height (volume) decreases, but experimental measurements of cell associated material made by Lison et al. [32] are roughly unchanged by media volume and height. The modeled trend is consistent with expectations: at extremely low volumes and media heights, all particles would be in immediate proximity to cells and be delivered rapidly and more completely, whereas at large volumes and media heights, only a fraction of particles would reach cells over the course of an experiment. Because cell associated silica was relatively constant across all experiments, where total particle number and media heights varied, it is also plausible that the uptake of particles by cells in the experiments by Lison et al. [33] was saturated at the tested particle exposure levels.

#### Transport of nano and micron sized polystyrene spheres

Transport rates of carboxylated polystyrene particles 24, 100, 210, 500 and 1100 nm in diameter were experimentally determined using time-lapse fluorescence microscopy (Axiovert, Zeiss) at TIRF configuration. For these constant particle number exposures, measured rates of particle transport varied approximately two-fold across particle sizes (Table 1). In comparison, modeled transport rates varied approximately three-fold with the highest transport rate predicted for the 20 nm particles, consistent with their rapid diffusional transport. Generally, there was good correspondence between measured and simulated rates of transport (Table 1). Differences, measured as the ratio of simulated to measured rates of transport varied from 0.37 to 0.51, a factor of 2-3.

**Table 1:** Comparison of observed and simulated transport rates of carboxylated polystyrene particles

**Particle Diameter (nm)**	**Transport Rate Particles/500 seconds**		**Simulated/Observed**
		
	**Observed^1^**	**Simulated**	
	
24	190	181	0.95
100	196/155	89	0.51
210	101/140	63	0.52
500	96/130	73	0.65
1100	140/200	62	0.37

1 Results from two experiments shown

#### Sedimentation of large iron oxide agglomerates

**Figure 4:**
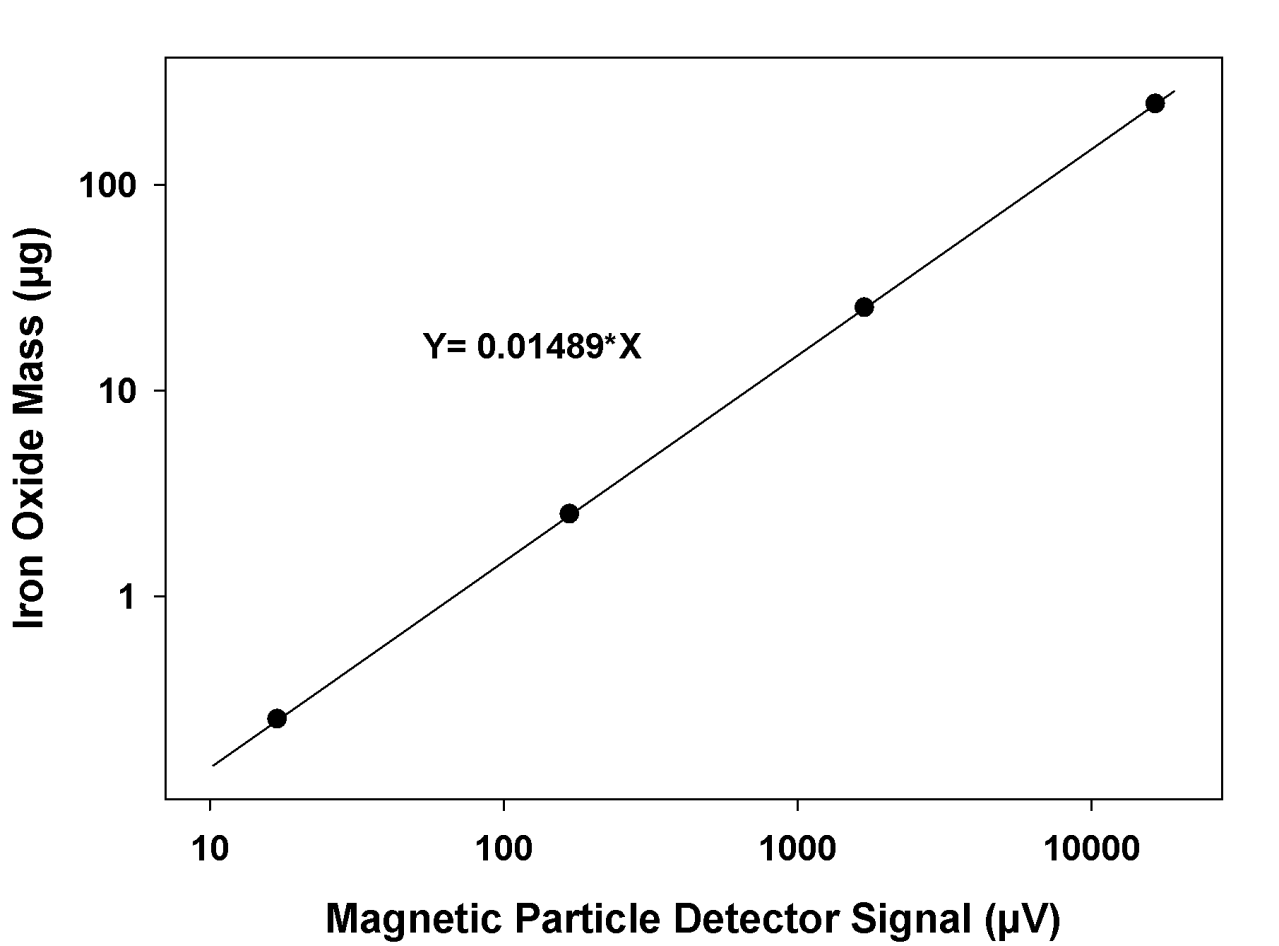
**Iron Oxide MPD Standard Curve**. Standard curve for analysis of iron oxide nanoparticles in RAW 264.7 macrophages showing linear responses across a more than 100 fold increase in particle mass.

The rate of transport of agglomerates of ~30 nm iron oxide particles was experimentally determined by measuring the amount of cell associated iron oxide in RAW 264.7 cells after 2, 4 and 8 hours of exposure. The standard curve for the magnetic particle detection system was highly linear across two orders of magnitude (Figure 4).

**Figure 5:**
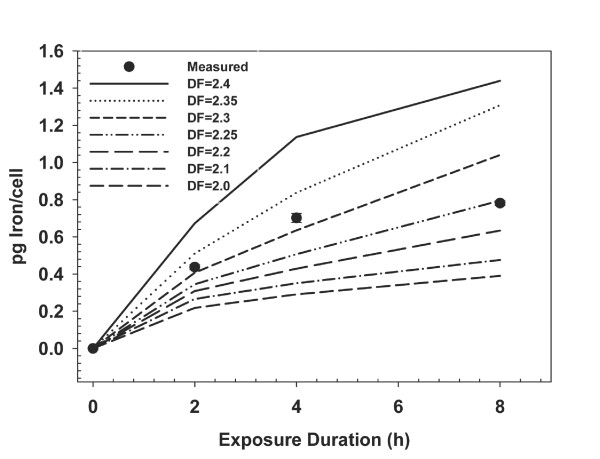
**Simulated Transport of Iron Oxide Agglomerates**. Comparison of the modeled and observed transport kinetics of iron oxide agglomerates to RAW 264.7 macrophages. Particle transport was modeled for plausible values of the agglomerate fractal dimension (DF); a DF of 2.3 provided the best correspondence between modeled and observed data. Error bars (visible only on one point) for experimental data reflect standard deviations. The media height was 1.06 mm in this experiment.

Transport of the polydisperse solution of agglomerates was modeled as a mass weighted distribution of particles in accordance with the particle size and number fraction associated with each agglomerate size class (Figure 2): The total mass of iron oxide delivered at each time step was calculated as the sum of calculated mass (fraction delivered times mass in the experiment) of each of the five agglomerate particle size classes, delivered. The fractal dimension (DF) of the iron oxide agglomerates was not known. Improbable values of DF, 1 (representing a rod) and 3 (representing a perfectly filled sphere) were not considered. Values near those reported for cerium oxide and fumed silicon dioxide of "around 2" [4,41] were considered plausible. Accordingly, DF was varied between 2.0 and 2.4, to evaluate model behavior against the experimental data. For this range of plausible values of DF, ISDD calculated delivered iron oxide was in close agreement with measured values of cell associated iron oxide (Figure 5), differing at most by a factor of approximately 2 or less. Correspondence between observed and model calculated transport of iron oxide was greatest, differing by only 5-30%, for a DF of 2.3. Of the values of DF tested, 2.3 was the most plausible value, consistent with the limited information available on DF for metal oxide particles. The formulation of the sedimentation velocity used in ISDD is one of two forms cited by Sterling [31]. The alternative formulation (Sterling[31], Table 1) assumes that media can flow through the agglomerate and uses the density of the primary particle rather than the density of the agglomerate. Resulting sedimentation rates would be 3-4 times faster than we observed experimentally for iron oxide particles and we therefore elected not to use this form of the aggregate sedimentation rate equation.

### Generalized Affect of Media Height, Particle Size and Density on Delivered Dose for Submicron Particles

Of the commonly varied experimental conditions or particle characteristics, media height, particle density, particle size, and agglomeration state are expected to have the greatest impact on the rate and extent of nanoparticle and microparticle transport, and thus delivered dose to cells *in vitro*. Using ISDD, the impact of these parameters on target cell dosimetry can be described quantitatively, providing a clearer portrait of the importance of these processes to both experimental design and interpretation. Specifically, these descriptions quantify the errors associated with ignoring the kinetics of particles in solution when conducting toxicity or dose-response studies.

#### Particle size and density

**Figure 6:**
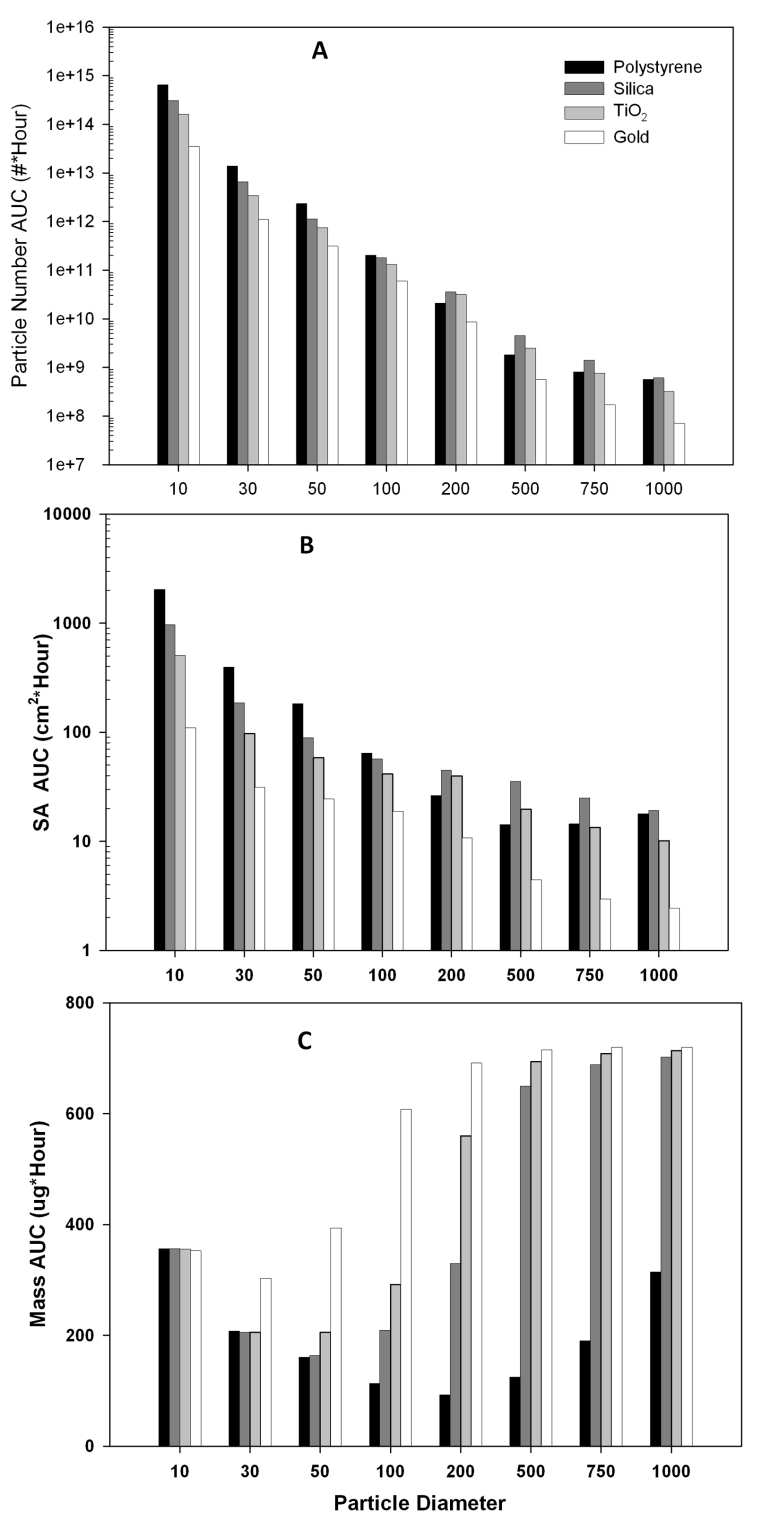
**Particle Size and Density Effects on Target Cell Dose**. Target cell doses calculated using ISDD for cells exposed 24 hours *in vitro *to 10 μg/mL (3 mL, media height 3.1 mm) of particles with different sizes and densities. Panels A, B and C present target cell AUC on a particle number, surface area and mass basis.

**Figure 7:**
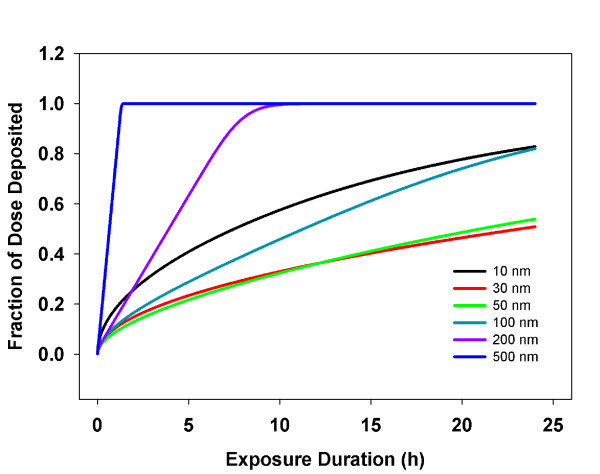
**Transport Rates for TiO_2_**. ISDD calculated fraction of nano and micron scale TiO_2 _particles delivered to cells over the duration of a 24 hour *in vitro *study with a media height 3.1 mm. Different rates of particle transport result in different time-courses for delivery to cells, which is only complete for large particles by 24 hours.

When the toxicity of nanoparticles or microparticles of different density and size are assayed *in vitro *using equivalent nominal media concentrations (e.g. 1-100 μg/mL), there are two factors which, depending on the dose metric of interest, may result in significant differences in target cell doses for each particle. First, the same nominal mass media concentration represents different particle numbers and different surface areas for each particle. Secondly, each particle may have a different transport rate. There is therefore no simple, consistent relationship between nominal particle exposures on a mass basis *in vitro*, and the corresponding cellular doses on a mass, surface area or particle number basis. This relationship is illustrated in Figure 6 which shows the ISDD-calculated cellular dose of particles across size and density (polystyrene to gold) on a particle mass, particle surface area, and particle number basis. To demonstrate the integrated effects of different transport rates on target cell doses, the time weighted target cell dose (area under the curve - AUC) was calculated using ISDD for a 10 μg/mL (3 mL media) exposure over a 24 hour exposure period (Figure 6). On a particle number basis, the target cell AUC varies six orders of magnitude across particle size and density (Figure 6A). On a surface area basis, the most commonly cited relevant metric of dose, target cell doses vary three orders of magnitude across particle size and density and approximately one order of magnitude between particles of the same size but different density (Figure 6B). A plot of the target cell dose on a particle mass basis shows smaller differences between particle of the same size and across particle size and density, but shows the expected U-shape for the effects particle size arising from the interplay between diffusive transport and sedimentation (Figure 6C). These differences in dose arise from differences in the rate and extent of particle transport over the duration of the experiment. Figure 7 shows the time course for particle transport for different sizes of TiO_2_, a representative metal oxide. The principle holds for particles with different densities.

The differences (5% to two-fold) between ISDD-estimated doses and experimentally measured cell dose across a wide range of particles with disparate size and density are quite small. Thus, ISDD supports comparison of biological response across diverse particles sizes and density by providing an accurate estimate of delivered dose.

In contrast, the difference between assumed dose based on equivalent exposures as μg/mL and the delivered dose can be several orders of magnitude when particles with different densities and sizes are compared. Two factors contribute to the sometimes very large differences (a) differences in size- and density-dependent transport rates and (b) differences in the number and surface area of particles per unit mass particle concentration. When particles of a similar size and density are compared, transport related effects on delivered dose may be small: 2-10 fold unless confounded by agglomeration. However, when the cellular dose for particulates of different size and density is assumed to be equivalent based on equivalent exposures (media concentration), the difference between assumed and actual delivered doses can differ by one to six orders of magnitude. ISDD offers significant advantages over the use of mass based metrics of nanomaterial exposure or assumptions of dose-equivalency based on metrics of exposure (e.g. μg/mL) for dose-response assessment.

#### Media height

**Figure 8:**
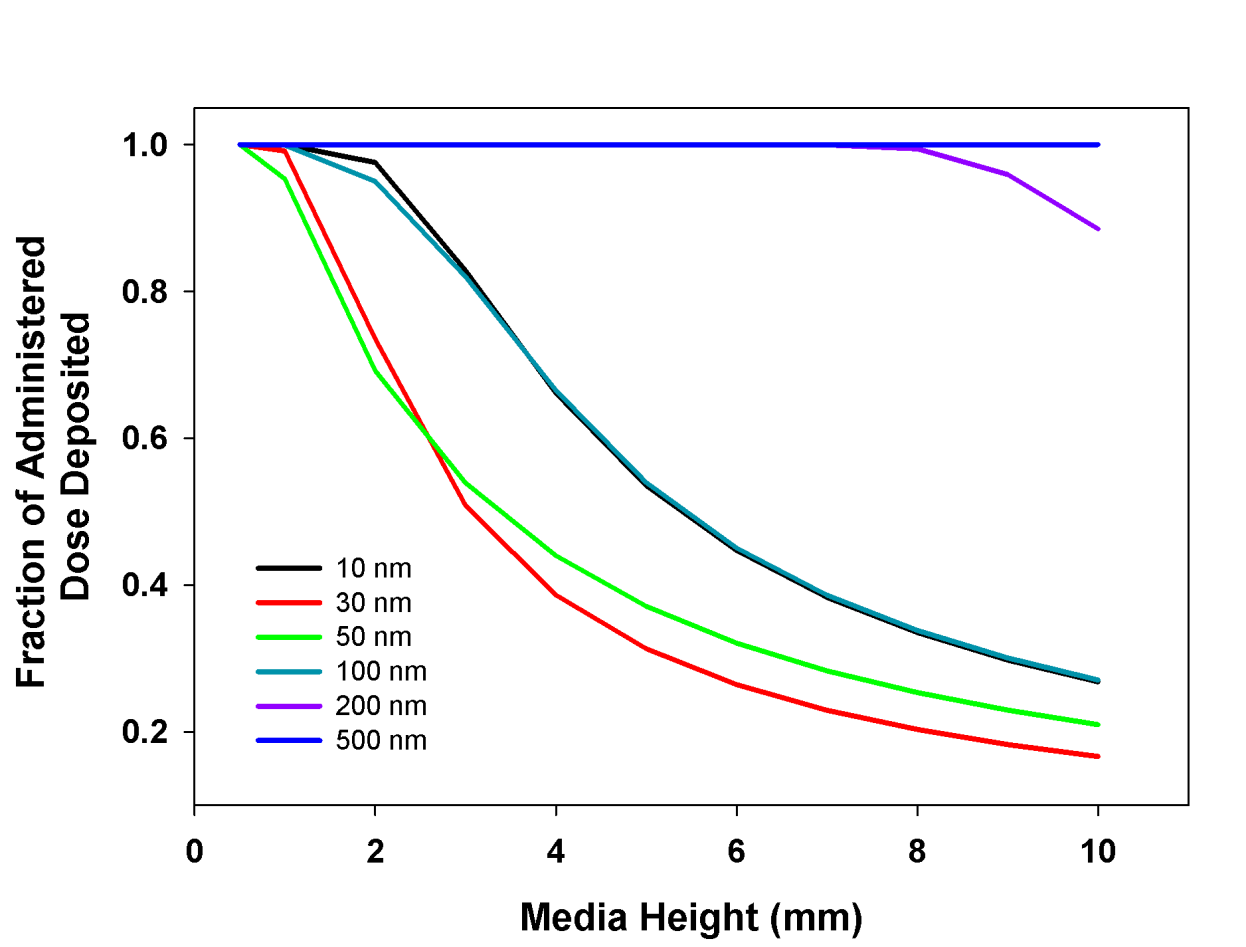
**Media Height Effects on the Extent of Transport**. ISDD calculated fraction of nano- and micron scale TiO_2 _particles delivered to cells over the duration of a 24 hour *in vitro *study as a function of media height. Increases in media height reduce the fraction of the administered dose reaching cells, particularly for nanoparticles, where diffusion drives transport.

Media height above cells in culture determines the distance particles must travel to reach cells and the total number of particles available for transport to cells. Particles distributed evenly at the start of an *in vitro *experiment will have a distribution of distances to travel (i.e. zero to media height), and a corresponding distribution of times to reach cells. At commonly used media heights (1-10 mm) and study durations (~24 hours), particle transport is neither constant nor fast enough to be non-limiting; rather, transport times are on the order of hours, similar to the duration of *in vitro *studies. For example, the extent of transport TiO_2 _particles 10-500 nm in diameter varies significantly with media height (Figure 8). An unexpected consequence of this relationship is that linear increases in total administered dose introduced through linear increases in media volume (constant concentration) bring a linear increase in media height, and consequently, a linear increase in the average distance a particle must travel to reach the cells. But, because the time to diffuse any distance is a function of the distance squared (Equation 6) [12,23], the linear increase in media height results in a quadratic increase in the transport time and non-linear--and at some media heights, saturating--transport of particles to the cells. Thus, increasing media volume and administered dose do not necessarily yield equivalent increases in target cell dose. Moreover, differences in media height alone between studies can confound comparisons of dose-response data.

#### Agglomeration state

Agglomeration and aggregation of particulates in high ionic strength aqueous solutions such as cell culture media is common. Formation of large agglomerates increases the size and mass of particles in proportion to the number of particles in the agglomerate. Due to the entrapment of media within the open volume of the agglomerate, the density of an agglomerate is generally less than that of the primary particle. The parameter DF, the fractal dimension, describes the space filling efficiency of agglomerate (EQ 7) and along with the packing factor (PF, EQ 7), determines the density of the agglomerate. Thus, agglomeration dependent changes in mass, size and density all effect changes in the rates of agglomerate diffusional and gravitational transport.

**Figure 9:**
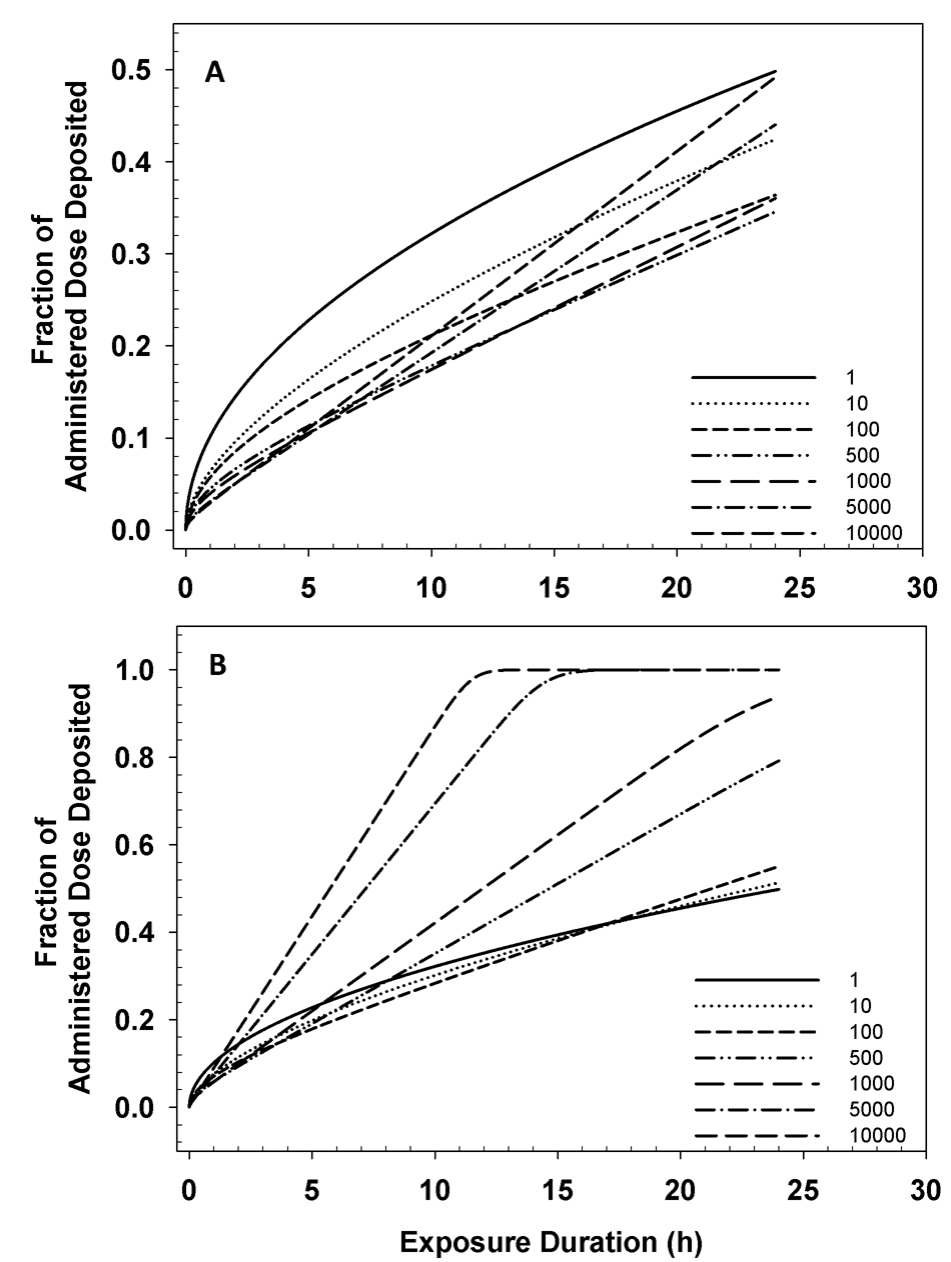
**Agglomerate Fractal Dimension Effects on Transport**. ISDD calculated rate of transport over a 24 hour *in vitro *exposure fto Fe_2_O_3 _agglomerates with, a primary particle size of 34.6 nm, and a fractal dimension of 2.2 (A) or 2.4 (B). The media height was 3.1 mm. Each line represents a different number of particles per agglomerate. Increasing the size and mass of agglomerates decreases diffusion rates, but may not increase sedimentation rates. For more efficiently packed agglomerates (DF = 2.4, bottom panel), but not less efficiently packed agglomerates, increases in agglomeration size can increase the rate and extent of sedimentation.

To demonstrate the importance of considering formation of agglomerates to target cell dosimetry, ISDD was used to simulate the transport of agglomerates of 1-10,000 primary 34.6 nm Fe_2_O_3 _particles over a period of 24 hours. To show the influence of packing efficiency, simulations were conducted for a fractal dimension of 2.2 or 2.4, the approximate range of DF determined in our simulations (Figure 9). Increasing the number of primary particles in a Fe_2_O_3 _agglomerate from 1 to 10,000 particles only increases sedimentation rates if their space filling efficiency is high (e.g. DF = 2.4, Figure 9B). Sedimentation rates decrease with agglomerate size when packing is less efficient (Figure 9A, DF = 2.2). These changes in the extent of sedimentation result from reduced agglomerate density.

Reductions in agglomerate density from inefficient packing can be significant, particularly where agglomerates are composed of a large number of primary particles (Additional File 1) or DF is large (Additional File 2). Large particles with a loosely packed structure can approach a density nearing that of the media as the volume and mass of trapped media dominates the total volume and mass of the agglomerate. Fractal dimensions between 2 and 2.5 are consistent with inefficient or moderately efficient space filling and agglomerates with a density significantly reduced from that of the monomer.

The relationship between agglomerate size, density and effective sedimentation rates depends on many factors (see EQ 7) and is therefore unique to each particle and exposure environment. The examples here demonstrate the potential effect and importance of agglomeration on particle dosimetry, but should not be applied to particles or systems different from the examples presented here.

## Discussion

Driven by wide recognition that target tissue (or cell) dose is the most appropriate metric of dose for risk and safety assessment, there has been continued growth in the development of computational tools for estimating target tissue dose *in vivo *for a wide range of materials: volatile and non-volatile materials [42-44] organic chemicals [45,46], pharmaceuticals [47,48], and particulates and ultrafine particulates [49-53]. It is surprising, then, given the growing importance of *in vitro *studies to chemical and particle risk assessment [10] that few efforts [43,54-56] have been directed at developing computational models of dosimetry for *in vitro *systems. In part, this oversight may be the result of the incorrect belief that in contrast to *in vivo*, there are no important kinetic or other processes to consider when addressing the issue of target tissue dose *in vitro*. We have shown this assumption is not universally true for chemicals [54] and particulates, including nanomaterials [12]. Thus, as with other systems where kinetics influence dose, there is a need for computational models of *in vitro *nanoparticle and microparticle target tissue dosimetry to supplement experimental measurements or extrapolate across particle type and experimental systems.

ISDD is a computational model of particokinetics (sedimentation, diffusion) and dosimetry for non-interacting spherical particles and their agglomerates in monolayer cell culture systems. Through simulation, ISDD calculates the delivered dose and rate of transport of particles *in vitro *using readily available parameters (temperature, media height, particle size in solution, agglomeration state and particle density). To our knowledge this is the first computational model extending principles long used to calculate particle deposition in the respiratory tract to a simpler system: static liquid *in vitro *studies. Application of ISDD opens the door for post-hoc interpretation of published studies, scaling across particle types and doses and development of a more predictive paradigm for nanoparticle *in vitro *studies.

Without adjustment of any parameters (calibration), ISDD simulated particle transport of monodisperse silica and polystyrene particles corresponded very well with measured transport, differing in most cases by a factor of approximately two or less. This level of accuracy is common to more sophisticated, extensively calibrated models of *in vivo *pharmacokinetics used in risk assessment, such as physiologically based pharmacokinetic (PBPK) models [46,57,58]. There cannot be an expectation that model accuracy is greater than limits imposed by experimental and biological variability, which can be significant. Thus, PBPK and other biokinetic models are commonly considered acceptable and useful if outputs are within a factor of approximately two (or sometimes more) of the data and the dose and time trends (model behavior) are consistent with experimentally measured trends. This general level of error can be compared to the other sources of error inherent in assuming mass media concentrations are, alone, sufficient measures of exposure to comparative toxicity studies for particles: 1) size and density differences in transport rates, approximately 2-10 fold; 2) media height differences in fraction of material transported to cells, up to approximately 10 fold; 3) particle size and density dependent differences in the number and surface area of particles per unit concentration (e.g. 1 μg/ml), 1,000-1,000,000 fold. Thus, the error in ISDD is low relative to the potential errors associated with common assumptions applied to most *in vitro *particle toxicity studies.

Beyond the accuracy issue, ISDD allows users to explore expected trends in delivered dose to determine if delivery processes are potentially important. For example, ISDD predicted a six-fold increase in transport of 35 nm amorphous silica as media heights were reduced from 4.5 mm to 1.1 mm, but measured cell associated silica was relatively constant[33]. The experimental finding could be the result of either constant transport rates, or one or more other experimental factors affecting cellular uptake such as saturated uptake of silica. For example, it is plausible that lower levels of nutrients may have affected cell function, uptake of particles, or the number of cells. Since ISDD shows that significant differences in particle transport and delivered dose are expected, we arrive at the hypothesis that cellular uptake might be saturated. Arriving at this insight, and the experimentally testable hypothesis it produces, is not feasible if the experimentalist is unaware of differences in particle transport under the experimental conditions or incorrectly assumes static particle concentration is all one needs to know for dose-response assessment.

With calibration of the parameter DF, ISDD also simulated the transport of a dispersion of more complicated agglomerates of 30 nm iron oxide particles, providing greater confidence that the generalization of particle transport represented by the model can be widely applied to nanoparticle and microparticle solutions including agglomerates. Large errors in model predictions were not observed, with one exception (low volume, silica transport experiment), providing good evidence that one or more of the simplifying assumptions used in the formulation of the model were not violated.

There are additional published experimental data that support the general accuracy of ISDD. Limbach and Stark were the first to carefully explore the relationship between particle size and delivered dose to cells *in vitro*, firmly and convincingly establishing that particle size and agglomeration state has a significant impact on dose to the cell [4]. Using ceria particles with mean diameters of 20-50 nm or 250-400 nm they exposed lung fibroblasts and measured ceria content by ICP-MS at 6-10 times over the 300 minute experiment. Comparing cellular ceria content and calculated ceria transport rates they showed that cellular ceria uptake of 20-50 nm particles was consistent with diffusion limited transport, and uptake of 250-400 nm particles was primarily controlled by sedimentation. They also showed that for a constant nominal mass media concentration the mass of ceria in cells was related to particle size, with greater mass amounts of ceria but smaller surface area and number concentrations found in cells exposed to larger, more rapidly sedimenting particles. The authors concluded that particle size was the most important factor determining the amount of material on or in cells and that particle concentration and total surface area were of "minor" importance. Limbach and Stark's findings [4] demonstrate the appropriateness of the Stokes-Einstein equation for predicting transport *in vitro*, and the importance of addressing these transport issues *in vitro *nanomaterial toxicity studies.

These findings were further verified by Sun et al. [59], in an elegant quantitative study of *in vitro *transport and uptake of fluorescently labeled 100 nm mesoporous silica nanoparticles in human lung cancer cells. The movement and uptake of particles was observed using differential interference contrast microscopy. Transport to the cell through the cell culture media was driven by diffusion. The diffusion rate calculated directly from measured rates of particle movement was 2.9 × 10^-8 ^cm^2^/s, in very close agreement with the theoretical value calculated from the Stokes-Einstein equation as applied in ISSD: 4.4 × 10^-8 ^cm^2^/s [59]. Not surprisingly, the diffusion rate slowed an order of magnitude as the nanoparticles neared the cell surface. It should also be noted that the most common approach to measuring particle size, DLS, directly measures the diffusivity of particles in solution and infers the size of the particle using the Stokes-Einstein equation. Thus, it would seem, that if one believes DLS instruments are accurate, belief that that the ISDD calculated diffusional transport rate is accurate should follow. Finally, we point out that the description of particle motion in liquids derived by Navier, Stokes and Einstein and applied in ISSD has been widely and successfully applied across multiple scientific and engineering disciplines for many decades.

There are, however, a number of limitations to be considered when using ISDD. Particle settling must not generate turbulence (low Reynolds numbers) and dynamic agglomeration or other particle interactions are not accounted for in the model. The model may not be appropriate to apply where advection occurs in the cell culture system or where there has been significant advective or mechanical mixing over the course of the experiment. Formulated for spheres or particles that can be adequately described as spheres, ISDD should not be used for fibers without additional modification and testing. Changes in the agglomeration or aggregation state of modeled particles would be expected to lead to larger discrepancies between modeled and observed target cell doses. Uncertainty in many of the parameters for the model is low; particle size, density, agglomeration state and media temperature and viscosity are easy to measure with sufficient accuracy. As noted, the PF and DF are more challenging to obtain experimentally, and represent an area of higher uncertainty. Nonetheless, ISDD provides an excellent approximation of the expected cellular dose as a function of particle size and density, and allows reasonable estimates of the range of errors introduced using metrics of exposure.

ISDD, now tested and verified, provides further quantitative evidence that use of nominal media concentration as a metric of "dose"--its actually exposure--confounds particle comparisons by introducing large errors from the assumption that dose to the target cell or site is proportional to media concentration across particle size, density, and agglomeration state. This erroneous assumption is particularly important where nominal mass media concentrations (μg/mL) are used for dose response analysis, but the biologically relevant dose-metric is target cell dose on a surface area or particle number basis. In this case, particle size and density dependent differences in transport rates are compounded by particle size and density dependent differences in particle number and surface area. Of course, this problem is somewhat mitigated by using nominal surface area concentrations in dose-response experiments. These conclusions, along with those regarding the influence of media height and agglomeration status on particle transport reaffirm the need for a far greater curiosity about target cell dosimetry *in vitro *and a correspondingly increased role for research on nanoparticle dosimetry for *in vitro *systems.

The value of organizing and conceptualizing the processes controlling particle transport and dosimetry in cell culture systems and presenting them in the form of a model to the community of biologists and other scientists using *in vitro *systems should not be overlooked. In the past, similar efforts such as the early publications on PBPK modeling and more recent biologically based dose-response (BBDR) models have led to a deeper and wider understanding of the systems being studied, enabled new biological or toxicological insights, and promoted more accurate study design and interpretation. Experimentalists can use ISDD to explore the potential impact of particle and media characteristics on target cell dose in their systems, and to guide experimental design. Hazard and risk assessors can utilize the model for post-hoc calculation of target cell doses from published studies. As more complete models of biological response to particles are developed, linkages to ISDD will allow inclusion of target cell dose-time vectors, improving the basis for biologically-based dose response analysis and predictive toxicology. Perhaps most importantly, the concepts represented by the ISDD model can be used to define a new paradigm for nanomaterial and particle dosimetry for *in vitro *systems that parallels the widely accepted paradigm for particle dosimetry *in vitro*. Absent now, such a paradigm would improve the accuracy and scalability of *in vitro *systems for hazard screening and exploratory mechanistic work.

The gold standard for particle dosimetry for *in vitro *nanotoxicology studies should be direct experimental measurement of the cellular content of the studied particle. However, where such measurements are impractical, unfeasible, and before such measurements become common, particle dosimetry models such as ISDD provide a valuable, immediately useful alternative, and eventually an adjunct to such measurements. The model also allows researchers to estimate trends in particle transport to determine if transport processes may be an important factor in the study. Ultimately, ISDD is a computational framework for describing particle transport that can raise awareness of particokinetic issues in vitro, and be revised to improve its accuracy for specific particles and linked to models describing cellular processes affecting uptake of particles.

## Competing interests

The authors declare that they have no competing interests.

## Authors' contributions

JGT designed the research program, designed experiments, conducted simulations and wrote the manuscript. PMH developed the computational model. GO, WBC and KRM designed and conducted experimental kinetic studies. BDT and JGP contributed to the experimental design, interpretation and manuscript preparation. All authors have read and approved the manuscript for publication.

## Supplementary Material

Additional File 1 **Figure S1: Agglomerate density as a function of the number of monomers in the particle**. This file contains a graph of the density of agglomerates as a function of the number of monomers within the agglomerate. Click here for file

Additional File 2 **Figure S2: Agglomerate density as a function of the agglomerate fractal dimension**. This file contains a graph of the density of agglomerates as a function of the fractal dimension of the agglomerate. Click here for file

## References

[B1] Methner M Hodson L Geraci C Nanoparticle emission assessment technique (NEAT) for the identification and measurement of potential inhalation exposure to engineered nanomaterials--part A J Occup Environ Hyg 2010 7 127 132 10.1080/15459620903476355 20017054

[B2] Oberdorster G Safety assessment for nanotechnology and nanomedicine: concepts of nanotoxicology J Intern Med 2010 267 89 105 10.1111/j.1365-2796.2009.02187.x 20059646

[B3] Holsapple MP Lehman-McKeeman LD Forum series: research strategies for safety evaluation of nanomaterials Toxicol Sci 2005 87 315 10.1093/toxsci/kfi286 16144823

[B4] Limbach LK Li Y Grass RN Brunner TJ Hintermann MA Muller M Gunther D Stark WJ Oxide nanoparticle uptake in human lung fibroblasts: effects of particle size, agglomeration, and diffusion at low concentrations Environ Sci Technol 2005 39 9370 9376 10.1021/es051043o 16382966

[B5] Waters KM Masiello LM Zangar RC Tarasevich BJ Karin NJ Quesenberry RD Bandyopadhyay S Teeguarden JG Pounds JG Thrall BD Macrophage responses to silica nanoparticles are highly conserved across particle sizes Toxicol Sci 2009 107 553 569 10.1093/toxsci/kfn250 19073995 PMC2639757

[B6] Puzyn T Leszczynska D Leszczynski J Toward the development of "nano-QSARs": advances and challenges Small 2009 5 2494 2509 10.1002/smll.200900179 19787675

[B7] Garcia I Munteanu CR Fall Y Gomez G Uriarte E Gonzalez-Diaz H QSAR and complex network study of the chiral HMGR inhibitor structural diversity Bioorg Med Chem 2009 17 165 175 10.1016/j.bmc.2008.11.007 19026553

[B8] Ruiz P Faroon O Moudgal CJ Hansen H De Rosa CT Mumtaz M Prediction of the health effects of polychlorinated biphenyls (PCBs) and their metabolites using quantitative structure-activity relationship (QSAR) Toxicol Lett 2008 181 53 65 10.1016/j.toxlet.2008.06.870 18662755

[B9] Ashek A Lee C Park H Cho SJ 3 D QSAR studies of dioxins and dioxin-like compounds using CoMFA and CoMSIA Chemosphere 2006 65 521 529 10.1016/j.chemosphere.2006.01.010 16487571

[B10] NRC Toxicity Testing in the 21st Century: A Vision and a Strategy 2007 Washington, DC: National Academies of Sciences

[B11] Walker NJ Bucher JR A 21st century paradigm for evaluating the health hazards of nanoscale materials? Toxicol Sci 2009 110 251 254 10.1093/toxsci/kfp106 19468057 PMC2708598

[B12] Teeguarden JG Hinderliter PM Orr G Thrall BD Pounds JG Particokinetics in vitro: dosimetry considerations for in vitro nanoparticle toxicity assessments Toxicological Sciences 2007 95 300 312 10.1093/toxsci/kfl165 17098817

[B13] Hardman J Limbird L (Eds.) Goodman & Gilman's The Pharmacological Basis of Therapeutics 2001 New York: MCGraw-Hill

[B14] Treinen-Moslen M Klaassen K Toxic Responses of the Liver Casarett and Doul's Toxicology: The Basic Science of Poisons 2001 New York: McGraw-Hill 471 489

[B15] Brown JS Wilson WE Grant LD Dosimetric comparisons of particle deposition and retention in rats and humans Inhal Toxicol 2005 17 355 385 10.1080/08958370590929475 16020034

[B16] Schroeter JD Kimbell JS Bonner AM Roberts KC Andersen ME Dorman DC Incorporation of tissue reaction kinetics in a computational fluid dynamics model for nasal extraction of inhaled hydrogen sulfide in rats Toxicol Sci 2006 90 198 207 10.1093/toxsci/kfj072 16344266

[B17] Witschi H Last J Klaassen K Toxic Responses of the Respiratory System Casarett and Doul's Toxicology: The Basic Science of Poisons 2001 New York: McGraw-Hill 515 534

[B18] NRC Science and Judgement in Risk Assessment 1994 Washington, DC: National Academy Press

[B19] Dankovic D Kuempel E Wheeler M An approach to risk assessment for TiO2 Inhal Toxicol 2007 19 Suppl 1 205 212 10.1080/08958370701497754 17886069

[B20] Sayes CM Wahi R Kurian PA Liu Y West JL Ausman KD Warheit DB Colvin VL Correlating nanoscale titania structure with toxicity: a cytotoxicity and inflammatory response study with human dermal fibroblasts and human lung epithelial cells Toxicol Sci 2006 92 174 185 10.1093/toxsci/kfj197 16613837

[B21] Warheit DB Sayes CM Reed KL Nanoscale and fine zinc oxide particles: can in vitro assays accurately forecast lung hazards following inhalation exposures? Environ Sci Technol 2009 43 7939 7945 10.1021/es901453p 19921917

[B22] Hussain SM Hess KL Gearhart JM Geiss KT Schlager JJ In vitro toxicity of nanoparticles in BRL 3A rat liver cells Toxicol In Vitro 2005 19 975 983 10.1016/j.tiv.2005.06.034 16125895

[B23] Mason M Weaver W The Settling of Small Particles in a Fluid Phys Rev 1924 23 412 426 10.1103/PhysRev.23.412

[B24] Anjilvel S Asgharian B A multiple-path model of particle deposition in the rat lung Fundam Appl Toxicol 1995 28 41 50 10.1006/faat.1995.1144 8566482

[B25] Elimelech M Gregory J Jia X Williams R Particle Deposition and Aggregation - Measurement, Modelling and Simulation 1995 Elsevier

[B26] Dusenberry DB Living at the Micro Scale: The Unexpected Physics of Being Small 2009 Cambridge, MA: Harvard University press

[B27] Bird RB Stewart WE Lightfoot EN Transport Phenomena 1960 John Wiley & Sons Inc

[B28] Kuusela E Steaty-State Sedimentation of Non-Brownian Particles with Finite Reynolds Number 2005 Helsinki University of Technology, Espoo, Finland

[B29] Davis RH Acrivos A Sedimentation of Noncolloidal Particles at Low Reynolds-Numbers Annual Review of Fluid Mechanics 1985 17 91 118 10.1146/annurev.fl.17.010185.000515

[B30] Kato H Suzuki M Fujita K Horie M Endoh S Yoshida Y Iwahashi H Takahashi K Nakamura A Kinugasa S Reliable size determination of nanoparticles using dynamic light scattering method for in vitro toxicology assessment Toxicol In Vitro 2009 23 927 934 10.1016/j.tiv.2009.04.006 19397995

[B31] Sterling MC Bonner JS Ernest AN Page CA Autenrieth RL Application of fractal flocculation and vertical transport model to aquatic sol-sediment systems Water Res 2005 39 1818 1830 10.1016/j.watres.2005.02.007 15899280

[B32] Xiong Y Shi L Chen B Mayer MU Lower BH Londer Y Bose S Hochella MF Fredrickson JK Squier TC High-affinity binding and direct electron transfer to solid metals by the Shewanella oneidensis MR-1 outer membrane c-type cytochrome OmcA J Am Chem Soc 2006 128 13978 13979 10.1021/ja063526d 17061851

[B33] Lison D Thomassen LC Rabolli V Gonzalez L Napierska D Seo JW Kirsch-Volders M Hoet P Kirschhock CE Martens JA Nominal and effective dosimetry of silica nanoparticles in cytotoxicity assays Toxicol Sci 2008 104 155 162 10.1093/toxsci/kfn072 18400775

[B34] Weaver JB Rauwerdink AM Sullivan CR Baker I Frequency distribution of the nanoparticle magnetization in the presence of a static as well as a harmonic magnetic field Med Phys 2008 35 1988 1994 10.1118/1.2903449 18561675 PMC4108637

[B35] Krause H-J Wolters N Zhang Y Offenhäusser A Miethe P Meyer MHF Hartmann M Keusgen M Magnetic particle detection by frequency mixing for immunoassay applications Journal of Magnetism and Magnetic Materials 2007 311 436 444 10.1016/j.jmmm.2006.10.1164

[B36] Nikitin MPea Quantitative real-time in vivo detection of magnetic nanoparticles by their nonlinear magnetization Journal ofApplied Physics 2008 103 07A304-307A304-303

[B37] Nikitin PI Vetoshko PM Ksenevich TI New type of biosensor based on magnetic nanoparticle detection Journal of Magnetism and Magnetic Materials 2007 311 445 449 10.1016/j.jmmm.2006.10.1180

[B38] Gleich B Weizenecker J Tomographic imaging using the nonlinear response of magnetic particles Nature 2005 435 1214 1217 10.1038/nature03808 15988521

[B39] Weizenecker J Gleich B Rahmer J Dahnke H Borgert J Three-dimensional real-time in vivo magnetic particle imaging Physics in Medicine and Biology 2009 54 L1 L10 10.1088/0031-9155/54/5/L01 19204385

[B40] Minard KR Magnetic Particle Imaging 2010 2 Oxford: Elsevier

[B41] Bergna HE Roberts WO (Eds.) Colloidal Silica: Fundamentals and Applications 2006 Boca Raton, FL: CRC Press, Taylor and Francis Group

[B42] Clewell RA Merrill EA Gearhart JM Robinson PJ Sterner TR Mattie DR Clewell HJ Perchlorate and radioiodide kinetics across life stages in the human: using PBPK models to predict dosimetry and thyroid inhibition and sensitive subpopulations based on developmental stage J Toxicol Environ Health A 2007 70 408 428 10.1080/15287390600755216 17454566

[B43] Hack CE Covington TR Lawrence G Shipp AM Gentry R Yager J Clewell HJ A pharmacokinetic model of the intracellular dosimetry of inhaled nickel J Toxicol Environ Health A 2007 70 445 464 10.1080/15287390600870726 17454569

[B44] Timchalk C Kousba A Poet T An Age-Dependent Physiologically-Based Pharmacokinetic/Pharmacodynamic (PBPK/PD) Model for the Organophosphorus Insecticide Chlorpyrifos in the Preweanling Rat Toxicol Sci 2007 10.1093/toxsci/kfm119 17504771

[B45] Teeguarden JG Bogdanffy MS Covington TR Tan C Jarabek AM A PBPK model for evaluating the impact of aldehyde dehydrogenase polymorphisms on comparative rat and human nasal tissue acetaldehyde dosimetry Inhal Toxicol 2008 20 375 390 10.1080/08958370801903750 18302046

[B46] Teeguarden JG Deisinger PJ Poet TS English JC Faber WD Barton HA Corley RA Clewell HJ Derivation of a human equivalent concentration for n-butanol using a physiologically based pharmacokinetic model for n-butyl acetate and metabolites n-butanol and n-butyric acid Toxicol Sci 2005 85 429 446 10.1093/toxsci/kfi103 15703268

[B47] Bradshaw-Pierce EL Eckhardt SG Gustafson DL A physiologically based pharmacokinetic model of docetaxel disposition: from mouse to man Clin Cancer Res 2007 13 2768 2776 10.1158/1078-0432.CCR-06-2362 17473210

[B48] Germani M Crivori P Rocchetti M Burton PS Wilson AG Smith ME Poggesi I Evaluation of a basic physiologically based pharmacokinetic model for simulating the first-time-in-animal study Eur J Pharm Sci 2007 31 190 201 10.1016/j.ejps.2007.03.008 17481865

[B49] USEPA External Review Draft Nanotechnology White Paper 2005 1 134

[B50] Asgharian B Anjilvel S A multiple-path model of fiber deposition in the rat lung Toxicol Sci 1998 44 80 86 10.1093/toxsci/44.1.80 9720144

[B51] Dynamics TGoL Depostion and Retention Models for Internal Dosimetry of the Human Respiratory Tract Health Physics 1966 12 173 207 5916786

[B52] Stober W Morrow PE Hoover MD Compartmental modeling of the long-term retention of insoluble particles deposited in the alveolar region of the lung Fundam Appl Toxicol 1989 13 823 842 10.1016/0272-0590(89)90337-0 2620799

[B53] Tran CL Jones AD Cullen RT Donaldson K Mathematical modeling of the retention and clearance of low-toxicity particles in the lung Inhal Toxicol 1999 11 1059 1076 10.1080/089583799196592 10562697

[B54] Teeguarden JG Barton HA Computational modeling of serum-binding proteins and clearance in extrapolations across life stages and species for endocrine active compounds Risk Anal 2004 24 751 770 10.1111/j.0272-4332.2004.00473.x 15209943

[B55] Masson-Pevet MA Bleeker WK Besselsen E Treytel BW Jongsma HJ Bouman LN Pacemaker cell types in the rabbit sinus node: a correlative ultrastructural and electrophysiological study J Mol Cell Cardiol 1984 16 53 63 10.1016/S0022-2828(84)80714-2 6699918

[B56] Treijtel N Barendregt A Freidig AP Blaauboer BJ van Eijkeren JC Modeling the in vitro intrinsic clearance of the slowly metabolized compound tolbutamide determined in sandwich-cultured rat hepatocytes Drug Metab Dispos 2004 32 884 891 10.1124/dmd.32.8.884 15258115

[B57] Bois FY Statistical analysis of Clewell et al. PBPK model of trichloroethylene kinetics Environ Health Perspect 2000 108 Suppl 2 307 316 10807560 10.1289/ehp.00108s2307 PMC1637757

[B58] Merrill EA Clewell RA Robinson PJ Jarabek AM Gearhart JM Sterner TR Fisher JW PBPK model for radioactive iodide and perchlorate kinetics and perchlorate-induced inhibition of iodide uptake in humans Toxicol Sci 2005 83 25 43 10.1093/toxsci/kfi017 15509666

[B59] Sun W Fang N Trewyn BG Tsunoda M Lin VS Yeung ES Endocytosis of a single mesoporous silica nanoparticle into a human lung cancer cell observed by differential interference contrast microscopy Anal Bioanal Chem 2008 391 2119 2125 10.1007/s00216-008-2162-1 18488205

