# Supplementary figures and images for: ISDD: A computational model of particle sedimentation, diffusion and target cell dosimetry for in vitro toxicity studies
Source: Part Fibre Toxicol. 2010 Nov 30;7:36. doi: 10.1186/1743-8977-7-36 (PMC3012653; doi:10.1186/1743-8977-7-36)

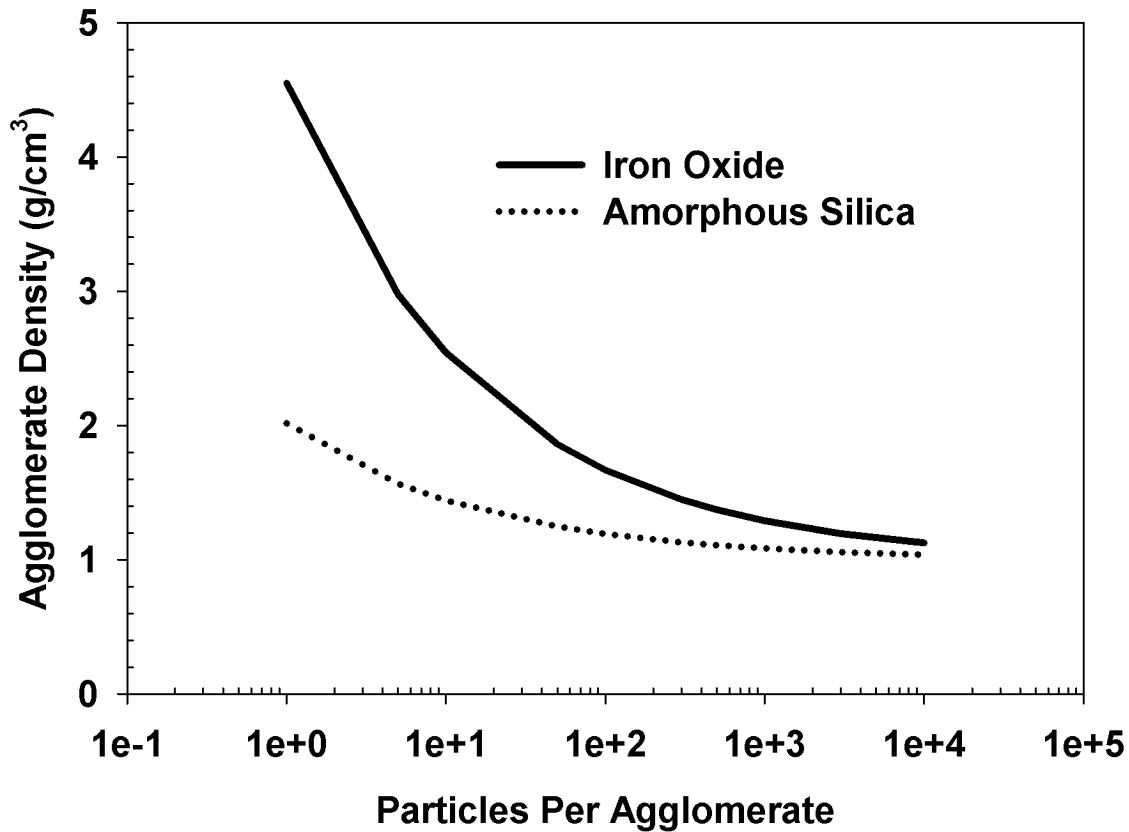

Supplement: Additional File 1 — Figure S1: Agglomerate density as a function of the number of monomers in the particle. This file contains a graph of the density of agglomerates as a function of the number of monomers within the agglomerate. [file 1743-8977-7-36-S1.PDF]

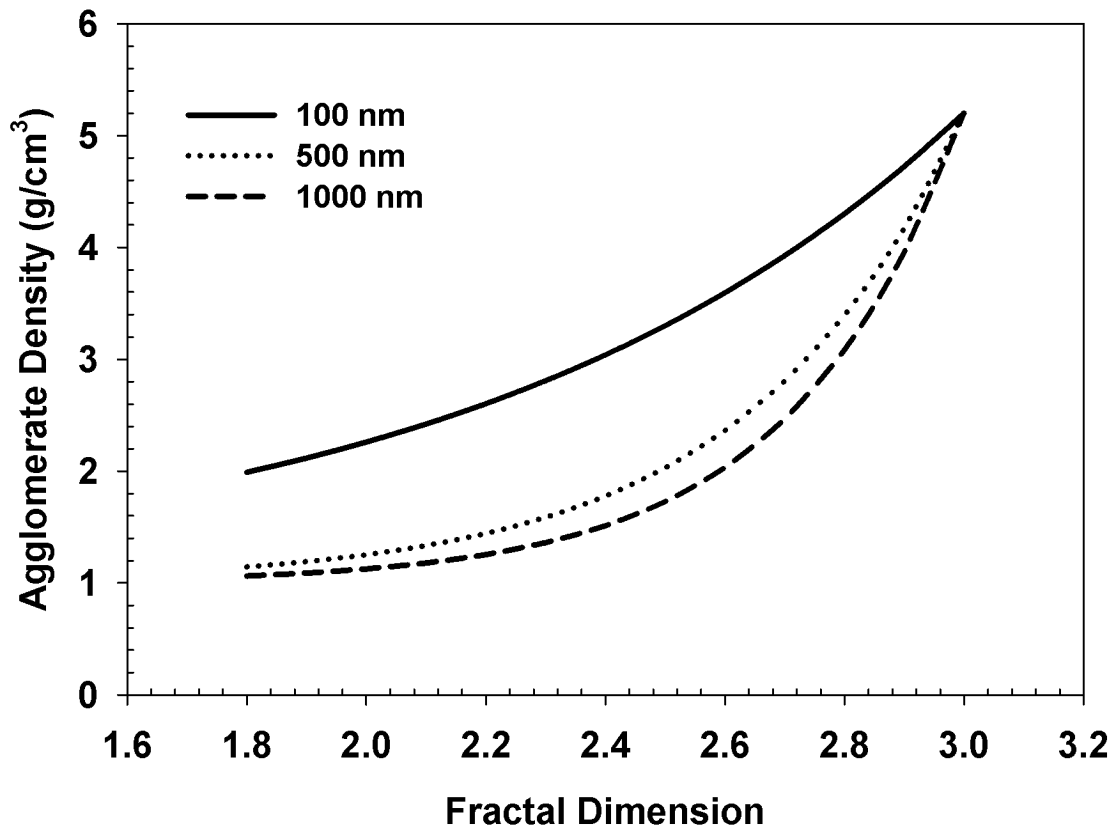

Supplement: Additional File 2 — Figure S2: Agglomerate density as a function of the agglomerate fractal dimension. This file contains a graph of the density of agglomerates as a function of the fractal dimension of the agglomerate. [file 1743-8977-7-36-S2.PDF]
